# Insertion torque *versus* mechanical resistance of mini-implants inserted in different cortical thickness

**DOI:** 10.1590/2176-9451.19.3.090-094.oar

**Published:** 2014

**Authors:** Renata de Faria Santos, Antonio Carlos de Oliveira Ruellas, Daniel Jogaib Fernandes, Carlos Nelson Elias

**Affiliations:** 1 Masters student of Orthodontics, Federal University of Rio de Janeiro, UFRJ.; 2 Associate professor, Federal University of Rio de Janeiro, UFRJ.; 3 PhD resident in Material Science, Military Institute of Engineering.; 4 Adjunct professor, Military Institute of Engineering.

**Keywords:** Orthodontic anchorage procedures, Torque, Orthodontics

## Abstract

**Objective:**

This study aimed to measure insertion torque, tip mechanical resistance to fracture and transmucosal neck of mini-implants (MI) (Conexão Sistemas de PróteseT), as well as to analyze surface morphology.

**Methods:**

Mechanical tests were carried out to measure the insertion torque of MIs in different cortical thicknesses, and tip mechanical resistance to fracture as well as transmucosal neck of MIs. Surface morphology was assessed by scanning electron microscopy (SEM) before and after the mechanical tests.

**Results:**

Values of mechanical resistance to fracture (22.14 N.cm and 54.95 N.cm) were higher and statistically different (P < 0.05) from values of insertion torque for 1-mm (7.60 N.cm) and 2-mm (13.27 N.cm) cortical thicknesses. Insertion torque was statistically similar (P > 0.05) to torsional fracture in the tip of MI (22.14 N.cm) when 3 mm cortical thickness (16.11 N.cm) and dense bone (23.95 N.cm) were used. Torsional fracture of the transmucosal neck (54.95 N.cm) was higher and statistically different (P < 0.05) from insertion torsional strength in all tested situations. SEM analysis showed that the MIs had the same smooth surface when received from the manufacturer and after the mechanical tests were performed. Additionally, no significant marks resulting from the manufacturing process were observed.

**Conclusion:**

All mini-implants tested presented adequate surface morphology. The resistance of mini-implants to fracture safely allows placement in 1 and 2-mm cortical thickness. However, in 3-mm cortical thickness and dense bones, pre-drilling with a bur is recommended before insertion.

## Abstract (pt)

**Objetivos:**

os objetivos foram determinar os torques de inserção, a resistência mecânica à fratura da ponta e do perfil transmucoso de mini-implantes (MI) de uma marca comercial (Conexão Sistemas de Prótese), e analisar sua morfologia de superfície.

**Métodos:**

foram realizados testes mecânicos para medir o torque de inserção dos MI em corticais de diferentes espessuras, também foi avaliada a resistência à fratura da ponta e do perfil transmucoso. Antes e após os ensaios mecânicos, foi avaliada a superfície dos MI no microscópio eletrônico de varredura (MEV).

**Resultados:**

os valores de resistência à fratura dos MI (22,14N.cm^2^ e 54,95N.cm^2^) foram maiores e estatisticamente diferentes (p < 0,05) dos torques de inserção nas corticais de 1mm (7,60N.cm^2^) e de 2mm (13,27N.cm^2^). Entretanto, o valor do torque de inserção na cortical de 3mm (16,11N.cm^2^) e no osso denso (23,95N.cm^2^) foi estatisticamente semelhante (p > 0,05) ao torque de fratura da ponta do MI (22,14N.cm^2^). O torque de fratura do perfil transmucoso (54,95N.cm^2^) foi maior e estatisticamente diferente (p < 0,05) dos torques de inserção em qualquer uma das situações testadas. A análise em MEV mostrou que os MI, como recebidos e após os testes mecânicos, possuem superfícies lisas e sem marcas significativas oriundas do processo de fabricação.

**Conclusão:**

os MI avaliados apresentaram adequada morfologia da superfície. A resistência do MI foi compatível com a instalação em corticais de 1 e 2mm, porém, em cortical de 3mm e em ossos densos recomenda-se prévia perfuração com broca.

## INTRODUCTION

Conventional intraoral anchorage (not supported by implants) might fail due to lack of rigidity of support structures or as a result of patient's noncompliance during treatment, especially with regard to the use of extraoral appliances. Therefore, since conventional anchorage systems present some limitations, whether biomechanical or with regard to patient's cooperation, the use of mini-implants (MI) is an excellent alternative in cases requiring maximum anchorage.^1^

Although MI may sometimes pose problems such as excessive clinical mobility, they are considered a safe, simple and low-cost anchorage method able to solve many difficulties related to anchorage. In order to improve stability, modifications have been made to screw design and surface treatment (sandblasting, acid-etching and microthreads).

In order to succeed in using a new product or a new technique, it is essential to test it. According to Barlow,^2^ the decision on whether to buy newly developed products or to use specific methods must be based on strong evidence of clinical efficacy, as well as on understanding the influence of these products over orthodontic treatment.

Insertion and removal of MI are simple procedures that can be performed by the orthodontist himself.^3^ They have a high success rate, between 84 and 92%,^4-7^ but are associated with certain risks, among which fracture is the most important. Mini-implant fracture normally occurs during the insertion procedure.^8^ A surgical intervention might be necessary to remove the fractured part when tooth movement is planned. Testing MI placement in different types of cortical bone is, therefore, of paramount importance to improve the safety of these devices.

The aim of the present study was to assess the surface morphology of MI after their insertion and removal in artificial bone, and to analyze their placement torque in different circumstances of cortical thicknesses, comparing these values with those of mechanical fracture resistance.

## MATERIAL AND METHODS

A total of ten mini-implants (1.5 mm in diameter, 6 mm in length, and transmucosal profile of 1 mm; Conexão(tm), São Paulo, Brazil) with a modified screw pitch (reduction in the threads interval in the cervical portion) were tested.

Surface morphology was analyzed by scanning electron microscopy at 20 kV (JEOL LSM-5800). MIs were placed in aluminum sample holders with the use of double-adhesive carbon tape. Each MI was observed under magnification of 25, 50 and 150 so as to check surface finishing, presence of machining defects or corrosion, and the shape of the tips and threads.

Mechanical tests were performed to measure the insertion torque and resistance to fracture of the MI tip and the transmucosal profile.

Insertion tests were carried out on the basis of ASTMF117 (Standard Test Method for Driving Torque of Medical Bone Screws) and F1622 (Standard Test Method for Measuring the Torsional Properties of Metal Bone Screw). The tests were performed in an EMIC DL 10.000 testing machine, with a 100 N load cell, displacement of 1 cm/min. Maximum placement and fracture torque values were recorded.

Mini-implants were inserted into blocks of polyurethane resin provided by Nacional Ossos.(tm) At the top of the block, resin with a density of 40 pcf (0.62 g/cm^3^) was applied to simulate cortical bone thicknesses of 1, 2 and 3 mm, whereas at the bottom portion, resin of 20 pcf (0.32 g/cm^3^) was applied to simulate cancellous bone. A 1-mm bur was used for perforation. The system consisted of two grips which forced the MI against the resin block (initial force of 400 gf) when compressed by an elastic force.

**Figure 1:**
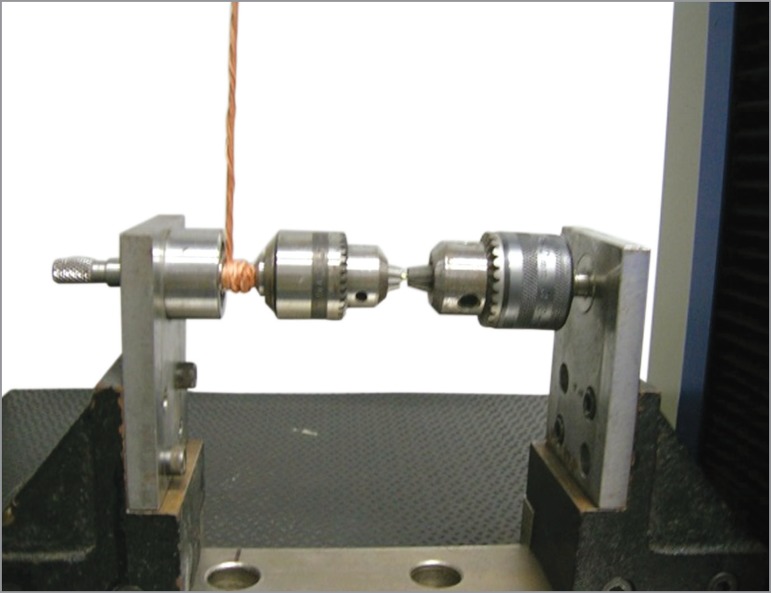
Device used for mini-implant torsional tests.

**Figure 2:**
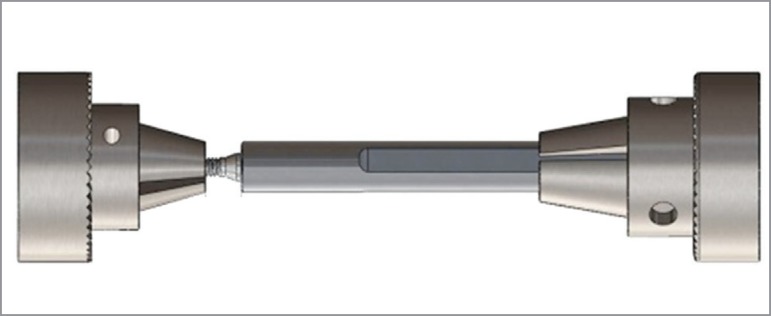
Approximate view of the device used for testing fracture torque, showing one MI attached to one grip and the insertion-removal key attached to other

To measure the fracture torque, mini-implants were attached to the devices shown in Figures 1 and 2. In the set up test, one of the grips was unable to rotate, while the other was free to move. To test the mechanical fracture resistance of the tip, the MI was inserted into the fixed grip up to the first three threads, while the insertion-removal key was secured in the other grip and attached to the head of the MI. To test the mechanical fracture resistance of the neck (transmucosal profile), the same system was used, with the difference that the MI was attached up to the second thread below the transmucosal profile. This testing device has an accuracy of 0.02 N.cm.

Data from all groups were analyzed using the SPSS for Windows software (v17.0; SPSS, Chicago, IL, USA). Means, standard deviations, medians, as well as minimum and maximum values were calculated. Normality and equality of variance of data were checked by the Kolmogorov-Smirnov test.

Results were statistically analyzed using one-way ANOVA with the Tukey's post-hoc test to detect differences among groups. P-value of < 0.05 was accepted as statistically significant.

## RESULTS

**Figure 3:**
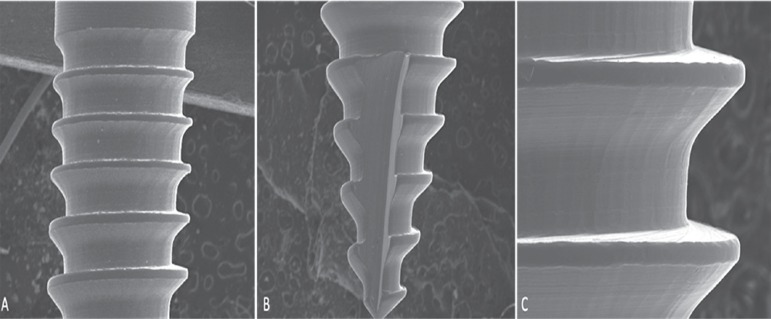
Photomicrographs of the mini-implants studied. A) Area next to the transmucosal profile where mechanical fracture resistance test was performed (x50); B) Area next to the tip where mechanical fracture resistance test was performed (x50); C) Image of mini-implant body (x150). Smooth surface without defects, and tip with a cutting area.

Figure 3 shows design modification (reduction in the distance between threads in the cervical portion), body and tip of mini-implants after insertion and removal. Photomicrographs of the mini-implants studied reveal smooth surface without defects, such as cracks and porosities, and without images that suggest corrosion. The tip presented a cutting area suitable for self-tapping.

Descriptive analysis of insertion and fracture torque values is shown in Table 1. MI tip fracture torque strength is lower than fracture torque strength of MI transmucosal profile.

**Table 1:** Insertion torque at different conditions, MIs resistence to fracture by torsion (N.cm^2^) and statistical analysis.

		Mean ± SD	Variation	Statistical difference*	Statistical difference*
Insertion torque	1 mm	7.60 ± 0.13	7.32 - 8.09	^B^ (P < 0.001)	^B^ (P < 0.001)
	2 mm	13.27 ± 0.34	12.30 - 13.89	^C^ (P = 0.003)	^C^ (P < 0.001)
	3 mm	16.11 ± 0.23	15.52 - 16.61	^A^ (P = 0.070)	^D^ (P < 0.001)
	dense bone	23.96 ± 0.11	23.50 - 24.11	^A^ (P = 0.949)	^E^ (P < 0.001)
Torque	Tip	22.14 ± 3.70	18.00 - 25.60	^A^	
Fracture	Transmucosal	54.95 ± 6.98	48.20 - 65.85		^A^

* Different letters in the same column mean statistical differences (P < 0.05).

## DISCUSSION

In Orthodontics, the use of mini-implants began with the purpose of providing anchorage and have proved to be an excellent alternative. The results have been so encouraging that these devices have been widely used.^9^

Surface morphology of the evaluated MI was similar to most MIs evaluated in the literature,^9,10,11^ presenting a smooth surface without significant damage resulting from the manufacturing process, and without signs of corrosion (Fig 3). Changes in morphology through surface treatment or mechanical modification might result in alterations in the growth and differentiation of osteoblasts.^12^ The presence of irregularities may increase osseointegration and, as a consequence, hinder MI removal. After insertion and removal, mini-implants showed no signs of deformation, even under high insertion torque (23.95 N.cm for dense bone).

Mini-implants can be placed in a wide range of sites,^13^ with the paramedian region of the palate, retromolar space, and especially interdental areas, being the most common sites. Areas between adjacent teeth normally present a cortical thickness of 1 or 2 mm.

As density and cortical thickness increase, it is necessary to drill the MI site. Edentulous areas, for example, require predrilling. For most locations where MI are placed (cortical thickness of 1 and 2 mm), the protocol is predrilling with a hand drill at the same place where the anesthesia puncture is performed. Cortical perforation with a hand drill ensures MI stabilization, which allows insertion into bone tissue. In the present study, the torque necessary to insert mini-implants into cortical thicknesses of 1 and 2 mm was lower than the torque that causes its tip to fracture (7.60 and 13.27 x 22.14 N.cm, respectively), thereby respecting a safety margin.

However, for cortical thickness of 3 mm and high density bones, such as edentulous areas, predrilling using a motor-driven handpiece at low rotational speed and under irrigation is necessary to minimize the risk of fracture. Although mini-implant placement under the aforementioned circumstances is less common, it might be the best indication for some clinical cases. The torque necessary to insert mini-implants into dense bone was higher than the fracture torque, although fracture did not occur during the placement procedure. This is probably associated with two facts: drill diameter (1 mm) close to the screw thread diameter where the fracture occurred (on the cutter), and the presence of this cutting area on the tip itself. Pithon et al^14^ observed that fracture torque strength is influenced by mini-implant shape. Design modification such as reduction in the distance between pitches, and the presence of a cutting tip, might consequently have influenced the values obtained.

Fracture torque strength values of the mini-implants assessed herein demonstrated that they are suitable for clinical use. It is important to emphasize, however, that MI placement in thick cortical or in dense bone requires predrilling with a drill. Moreover, the fracture torque strength values obtained in the present study were equal to or higher than those found in other studies using different brands of MI.^10,11,14,15,16^ This difference is probably associated with mini-implant shape, manufacturing process, and methodology used in this research.

The concern about fracture resistance is justified by the importance of performing a safe insertion procedure without risks of fracturing the mini-implant.^10,11^ Therefore, a MI with fracture torque strength higher than the torque necessary for insertion into bone tissue must be chosen. Although the incidence of fracture is not high (about 4%^17^), it might occur due to manufacturing process problems, professional mistake during placement, and - especially - application of excessive insertion torque. The latter is probably the reason for the lower success rate of mini-implants among beginners, since they commonly produce high torsional stress during placement.^3^ When using a mini-implant that has a fracture torque strength higher than its insertion torque, there is less chance of fracturing the device during placement, which could be demonstrated by placing it in cortical thicknesses of 1 and 2 mm. Consequently, the use of insertion key coupled to a torque gauge or with torque limiter, is recommended. The insertion torque values obtained in the present study suggested that torque be limited to a maximum of 15 Ncm. Should a higher insertion torque be required, the placement procedure must be stopped and predrilling with a drill must be performed. Cases in which predrilling has already been done, a drill with a larger diameter, approaching the diameter of the mini-implant, must be used. It is important to emphasize that this situation is not common and is normally restricted to old edentulous areas.

It is important to choose proper-sized transmucosal profile (neck) in accordance with the thickness of the soft tissue region where the MI will be installed, thus allowing the platform (region between the head and the transmucosal profile) to be well adapted to the soft tissue without causing excessive ischemia. Transmucosal profile greater than soft tissue thickness will create a gap between the platform and the soft tissue, thereby favoring bacterial plaque accumulation and leaving the MI in greater contact with the cheek or lip. It may also induce the professional to make a mistake because in order to bring the platform close to the soft tissue, he/she will try to insert part of the transmucosal profile in the bone, thus excessively increasing the insertion torque and the risk of fracture.

Stability achieved by mini-implant is one of the criteria used to evaluate the possibility of load application. The greater the primary stability is, the safer the clinical use of MI. In the present study, insertion torque values were higher than those normally found in the literature. This difference may be associated with the reduction in the distance between threads in the cervical portion, since it increases the number of threads and bone-implant contact area.

Although removal torque was not evaluated, it is normally lower than the insertion one. Cheng et al^4^ evaluated the removal torque of 46 MI removed from patients, and found values ranging from 10.78 to 21.07 N.cm. These values are lower than the fracture torque of MIs assessed in the present study.

## CONCLUSIONS

Mini-implants with microthreads presented smooth surface with proper finishing and without signs of deformation after insertion and removal.

Fracture resistance of the transmucosal profile was higher than the insertion torque in the different tested situations.

Fracture resistance of MI tip was compatible with its placement in cortical thicknesses of 1 and 2 mm. However, for 3 mm cortical and dense bones, predrilling is recommended.
